# The role of climate change education on awareness, anxiety, and hope among pregnant women: a randomized controlled study

**DOI:** 10.1186/s12884-026-09698-7

**Published:** 2026-07-25

**Authors:** Sevilay Aydın Çeli̇k, Beyzanur İşbay Aydemi̇r, Melike Di̇şsi̇z, Mine Akkuş Kala

**Affiliations:** 1 Cemil Taşcıoğlu City Hospital, Istanbul, Türkiye; 2 Faculty of Health Sciences, Department of Nursing, Istanbul Aydın University, Istanbul, Türkiye; 3 Hamidiye Faculty of Nursing, Department of Nursing, University of Health Sciences, Istanbul, Türkiye; 4 University of Health Science, Zeynep Kâmil Women and Children Diseases Traning and Research Hospital, Istanbul, Türkiye

**Keywords:** Anxiety, Climate Change, Health Education, Mental Health, Pregnancy

## Abstract

**Background:**

Climate change poses significant risks to physical and psychological health, particularly among vulnerable groups such as pregnant women. Awareness of climate-related threats can influence anxiety levels as well as coping mechanisms such as hope. This study aims to evaluate the effects of climate change education on pregnant women’s awareness, anxiety, and hope.

**Methods:**

This randomized controlled pretest–posttest experimental study was conducted with 154 pregnant women who attended a public hospital in Istanbul between September 2025 and January 2026. Participants were randomly assigned to either the intervention group (*n* = 77) or the control group (*n* = 77). The intervention group received a 60-minute face-to-face training session focusing on climate change and pregnancy, whereas no intervention was applied to the control group. Data were collected before the intervention and four weeks afterward using the Climate Change Worry Scale, the Climate Change Awareness Scale, and the Climate Change Hope Scale. The data were analyzed using descriptive statistics (such as frequency, percentage, and mean), parametric tests (independent samples t-test and ANOVA).

**Results:**

The mean age of participants in the intervention group was 28.49 ± 6.55 years, while it was 27.99 ± 6.85 years in the control group. No significant differences were found between the groups in terms of individual and obstetric characteristics (*p* > 0.05). Following the intervention, significant increases were observed in climate change anxiety, awareness, and hope levels in the intervention group (*p* < 0.001). In contrast, the control group showed decreases in anxiety and hope levels, while awareness demonstrated only a limited increase (*p* < 0.05). Between-group comparisons revealed that posttest scores were significantly higher in the intervention group (*p* < 0.05). Repeated measures ANOVA indicated a significant interaction between group and time for all variables (*p* < 0.001). Effect sizes were found to be large (d = 1.30–2.20), indicating a strong impact of the intervention.

**Conclusion:**

The findings of this study demonstrate that climate change education is an effective intervention that significantly influences awareness, anxiety, and hope levels among pregnant women. These results suggest that structured educational programs for pregnant women may serve as a valuable tool in enhancing environmental awareness and supporting psychological well-being.

**Trial registration number:**

NCT07403513, www.clinicaltrials.gov. Registered on January 29, 2026.

## Background

Climate change is widely recognized as one of the most significant global health threats of the 21st century, exerting multidimensional impacts not only on environmental systems but also on human health (1). Rising temperatures, increasing air pollution, food and water insecurity, and the growing frequency of extreme weather events negatively affect both physical and mental well-being. These impacts are particularly pronounced among vulnerable populations, with pregnant women considered one of the most at-risk groups due to their physiological and psychosocial characteristics (2). During pregnancy, immunological and hormonal changes heighten sensitivity to environmental stressors, thereby intensifying the potential risks of climate change for both maternal and fetal health (1, 2).

For many years, research on the effects of climate change during pregnancy has primarily focused on physical health outcomes. Exposure to extreme heat, air pollution, and environmental hazards has been linked to adverse outcomes such as preterm birth, low birth weight, and various obstetric complications (3). However, recent studies have increasingly emphasized the mental health consequences of climate change (1, 2, 4). Uncertainty, perceived threats, and concerns about the future associated with climate change can contribute to psychological distress, including stress, anxiety, and depression (4). During pregnancy, women often experience additional concern not only for their own health but also for the well-being of their unborn child, which may further intensify this psychological burden (1).

The concept of “climate anxiety” (also referred to as eco-anxiety or climate change worry) has gained prominence in the literature as a way to describe individuals’ emotional responses to climate change (5). Recent evidence suggests that climate anxiety is significantly associated with depression and anxiety in pregnant women and may even serve as a predictor of depressive symptoms (1, 4, 6). Nevertheless, climate-related anxiety does not exclusively lead to negative psychological outcomes; it may also foster a sense of hope and motivate individuals to engage in problem-solving and environmental action (6). This dual nature indicates that emotional responses to climate change can simultaneously involve both concern and hope (4, 6).

The psychological effects of climate change are not limited to direct exposure to environmental events but are also closely linked to how individuals perceive and interpret these changes (7). Studies have shown that, among pregnant women, climate change is associated with themes such as future-related anxiety, uncertainty, feelings of ecological loss, and psychological burden (1, 5, 8). However, existing research has largely focused on direct climate-related events, such as natural disasters, while perception-based studies remain limited (1, 7). This highlights the importance of investigating awareness and cognitive appraisal processes in this context (7).

In this regard, education emerges as a key intervention tool that can enhance individuals’ knowledge, awareness, and coping skills related to climate change (8, 9). Educational approaches help individuals structure their risk perceptions, better understand environmental threats, and develop more effective coping strategies (9, 10). Research indicates that climate change education can increase awareness, knowledge, and environmental perception among pregnant women while also reducing anxiety levels (2, 3, 8). Furthermore, such education has been shown to strengthen individuals’ sense of control, which in turn supports the development of hope (11).

Efforts to address climate change and its health-related consequences are closely aligned with the Sustainable Development Goals established by the United Nations. In particular, Goal 3 (Good Health and Well-being), Goal 5 (Gender Equality), and Goal 13 (Climate Action) are critically important for protecting pregnant women, supporting adaptation processes, and reducing health inequalities (12). Educational interventions developed within this framework can contribute not only to improving individual health outcomes but also to enhancing climate awareness at the societal level (9, 10).

Although current literature highlights the psychological effects of climate change on pregnant women, there is a notable lack of experimental studies examining the impact of educational interventions on awareness, anxiety, and hope. Therefore, evaluating the psychosocial outcomes of climate change education for pregnant women is an important necessity for both clinical practice and public health policy.

### Research hypotheses

- H_1_: After the educational intervention, participants in the intervention group will have lower climate change anxiety scores compared to the control group.
- H_2_: After the educational intervention, participants in the intervention group will have higher mean climate change awareness scores compared to the control group.
- H_3_: After the educational intervention, participants in the intervention group will have higher mean hope scores regarding the prevention of climate change compared to the control group.

## Methods

### Aim of the study

This study was conducted to determine the effects of climate change education for pregnant women on their levels of awareness, concern and hope.

### Research design

**Fig. 1:**
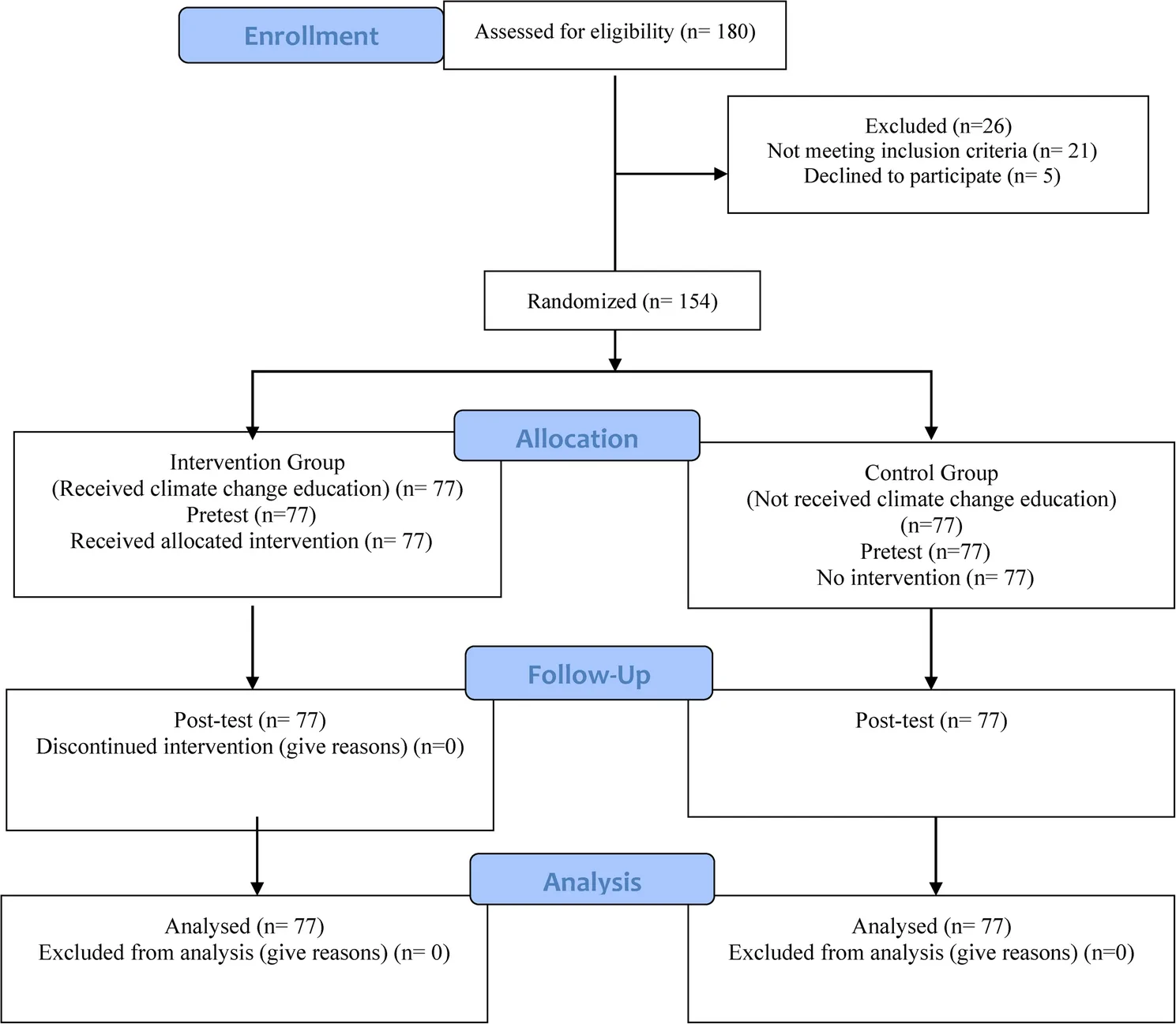
CONSORT flow diagram

The study was conducted between September 2025 and January 2026 using a randomised controlled experimental design with a pre-test–post-test control group, in accordance with the Consolidated Standards of Reporting Trials (CONSORT) guidelines (Fig. 1). The study protocol was established prior to the initiation of data collection. The study was registered in the ClinicalTrials.gov database under registration number NCT07403513 (Registration Date: 29 January 2026); the registration process had been initiated before data collection but was completed later due to administrative procedures.

### Setting and participants

The study was conducted at the antenatal clinic of a public hospital located on the Asian side of Istanbul. The hospital where the study was carried out is one of the region’s long-established institutions and provides comprehensive services in the fields of obstetrics and paediatrics. The study population consisted of pregnant women who attended the antenatal clinic for routine check-ups between September 2025 and January 2026.

### Sample size, randomization and allocation

The sample size was calculated using the G*Power 3.1 software. The power analysis was based on the t-test for independent samples; the effect size (Cohen’s d) was set at 0.50 (13), the significance level (α) at 0.05, and the statistical power (1-β) at 0.80. The analysis determined that a total of 128 participants, with at least 64 participants per group, would be sufficient for the study. To account for potential data losses, the sample size was increased by 20% in both the intervention and control groups, resulting in 77 participants in each group, and the study was completed with a total of 154 participants.

A total of 180 participants were assessed for eligibility. Of these, 26 were excluded; 21 did not meet the inclusion criteria (including the presence of a systemic disease before or during pregnancy or a history of pregnancy-related complications such as preeclampsia or gestational diabetes), and 5 declined to participate. The 154 participants who met the inclusion criteria and volunteered to participate were assigned to the groups using a simple randomisation method to prevent bias. The randomisation process was carried out using the web-based software www.randomizer.org. For this purpose, a random sequence of numbers between 1 and 154 was generated, and these numbers were distributed equally between the intervention and control groups (in a 1:1 ratio). The random allocation sequence was generated by an independent researcher, while participant enrolment and group assignment were carried out by the study researchers. The allocation sequence was concealed from the researchers enrolling participants until group assignment was completed. Given the nature of the educational intervention, blinding of participants and intervention providers was not possible. To reduce potential bias, outcome assessment and statistical analyses were conducted by individuals who were unaware of group assignments.

The intervention group received climate change education delivered individually to each participant in a private setting to minimize interaction between participants and reduce the risk of contamination between groups. A pre-test was administered to all participants prior to the intervention (*n* = 77). No intervention was administered to the control group; however, a pre-test was also conducted for participants in this group (*n* = 77).

### Inclusion and exclusion criteria

Inclusion criteria included being 18 years of age or older, having the ability to understand, speak, and read Turkish, being at or below 32 weeks of gestation at the time of enrollment, and providing voluntary consent to participate in the study.

Exclusion criteria included having a current psychiatric disorder or receiving psychiatric treatment, the presence of any systemic disease before or during pregnancy, and a history of pregnancy-related complications such as preeclampsia, gestational diabetes, or similar conditions.

### Study procedure and ethical considerations

Ethical approval (09.07.2025/84) was obtained from the Ethics Committee of the Zeynep Kâmil Women’s and Children’s Diseases Training and Research Hospital, where the study was conducted, and institutional authorisation (E-11391090-000-287000761 Date: 02.09.2025) was obtained from the hospital. In accordance with the Helsinki Declaration, the necessary explanations regarding the study (the purpose of the study, that participants’ names would be kept confidential, that data would be used for scientific purposes, and that they could withdraw from the study at any time) were provided to the pregnant women participating in the study, and their written and verbal consent was obtained.

The study was completed in two stages.

### Stage one: preliminary testing of the study

Participants in both the intervention and control groups were invited to take part in a study on climate change. The purpose and design of the study were explained to the participants, and assurances were given regarding voluntary participation, data confidentiality and the right to withdraw at any time. Informed consent forms were obtained prior to the data collection process. Subsequently, all participants completed the “Personal Information Form”, which collected sociodemographic data, as well as the “Climate Change Worry Scale (CCWS)”, the “Climate Change Hope Scale (CCHS)” and the “Climate Change Awareness Scale (CCAS)”.

### Stage two: intervention and final testing

Once the pre-test had been completed, participants in the intervention group received training on climate change and pregnancy, delivered face-to-face in a single session lasting approximately 60 min by the researcher, who is a nurse specialising in women’s health and nursing. To ensure the consistency and standardisation of the training presentation, the session was delivered using a standardised training content plan prepared by the researchers, presentation slides prepared in advance in line with the literature (1, 2, 3), and a brochure on the topic of ‘Climate change and pregnancy’. The training was delivered by the same researcher, and the duration and content of the session were kept consistent for all participants. A digital timer was used to monitor the duration of each training session. To ensure intervention fidelity, a standardized checklist was completed during every session. The checklist included verification of the planned session duration, delivery of all educational content, use of the standardized presentation slides and educational brochure, coverage of all planned topics related to climate change and pregnancy, and provision of recommendations regarding protective health behaviours. This procedure ensured that all participants received the intervention in a consistent and standardized manner. The training content included information on the definition of climate change, rising temperatures, rainfall irregularities, air pollution and the effects of natural disasters on human health. Furthermore, within the scope of the effects of climate change on pregnancy, the relationship between extreme heat and the risk of preterm birth, the effects of air pollution on maternal and foetal health, issues relating to water and food security, and the risks that natural disasters may pose during pregnancy were addressed. Recommendations were made regarding adequate fluid intake, protection from hot weather, avoiding factors that adversely affect air quality, a balanced diet, regular health check-ups and disaster preparedness, all aimed at protecting the health of mothers and babies. Finally, participants were informed about the options available to them for seeking assistance from family health centres, obstetricians and environmental health units regarding the educational and counselling services they might require during pregnancy. Similar studies have reassessed the effects of educational interventions using follow-up periods ranging from 3 to 6 weeks (1, 2, 3, 5, 7). In this study, a four-week follow-up period was also preferred in order to evaluate the short-term retention of the educational intervention and early changes in psychological outcomes.

### Data collection and outcome measures

The following instruments were used to collect the research data: the “Personal Information Form”, the “Climate Change Worry Scale (CCWS)”, the “Climate Change Awareness Scale (CCAS)”, and the “Climate Change Hope Scale (CCHS)”.

#### Personal information form

The questionnaire, prepared by the researcher based on the literature, consists of 18 questions designed to assess participants’ sociodemographic characteristics—such as age, educational status, spouse’s educational status, employment status, family type and income level—as well as their general health history and obstetric characteristics, including the number of pregnancies and whether the pregnancy was planned (1, 3, 4).

#### Climate change worry scale (CCWS)

The scale, developed by Stewart (2021), comprises 10 items and uses a five-point Likert-type rating scale. Participants rate each item on a scale of “1 - never”, “2 - rarely”, “3 - sometimes”, “4 - often”, and “5 - always” (14). A validity and reliability study for Turkey was conducted in 2021 by Gezer and İlhan, and the Cronbach’s alpha coefficient was found to be 0.91. Higher scores indicate an increase in the individual’s anxiety regarding climate change. The Turkish-adapted version consists of a two-factor structure comprising “anxiety” and “sense of helplessness”. Items 6, 7 and 9 are used to assess the sense of helplessness, whilst items 1, 2, 3, 4, 5, 8 and 10 are used to assess anxiety. The Cronbach’s alpha coefficients for “anxiety” and “sense of helplessness” are 0.87 and 0.83, respectively (15). In this study, the scale’s Cronbach’s alpha coefficient was calculated as 0.92.

#### Climate change awareness scale (CCAS)

The five-point Likert scale (ranging from ‘strongly disagree’ (1) to ‘strongly agree’ (5)), developed by Ataklı and Kuran (2022), consists of five factors and 52 items. For all items, 5 points are awarded for the ‘Strongly agree’ option, 4 points for ‘Agree’, 3 points for ‘Don’t know’, 2 points for ‘Disagree’ and 1 point for ‘Strongly disagree’. A score of between 52 and 260 can be obtained on this scale. Higher scores on the scale indicate a higher level of climate change awareness. The scale’s factors are: climate change awareness, perception of the problem, knowledge of the causes of climate change, climate change anxiety, and expectations regarding behaviours and policies. The Cronbach’s alpha value for the total scale is 0.92; 0.93 for ‘expectations regarding behaviour and policies’, 0.91 for ‘concern about climate change’, 0.87 for ‘knowledge of the causes of climate change’, 0.80 for “climate change awareness”, and 0.81 for “perception of the problem” (16). In this study, the Cronbach’s alpha coefficient for the scale was calculated as 0.93.

#### Climate change hope scale (CCHS)

The five-point Likert scale developed by Li and Monroe (2018) consists of a total of 11 questions (17). On this scale, participants are expected to rate each statement on a scale ranging from “1- Does not describe me at all” to “5- Describes me perfectly”. The scale, whose validity and reliability analysis in Turkish was conducted by Gezer and İlhan in 2020, is used to examine behavioural tendencies in the fight against climate change from various perspectives. The first three questions (1, 2, 3) assess the individual’s willingness and belief in finding solutions (BW); the fourth to eighth questions (4, 5, 6, 7, 8) assess the community’s willingness and belief in finding solutions (CW); whilst the final three questions (9, 10, 11) assess reluctance and hopelessness regarding finding solutions (U). Individual and societal willingness scores reflect hope that climate change can be prevented, whereas expressions of reluctance and hopelessness represent the opposite-that is, a hopeless outlook. For this reason, the scores for questions 9, 10 and 11 are treated as negative when calculating the total score of the scale. The Cronbach’s alpha value of the scale has been determined as 0.87 (18). In this study, the Cronbach’s alpha coefficient of the scale was calculated as 0.91.

### Statistical analysis

The data obtained in the study were analysed using SPSS (Statistical Package for the Social Sciences) version 25.0. Descriptive statistics were presented as mean ± standard deviation for continuous variables and frequency (percentage) for categorical variables. Independent samples t-test was used to compare continuous sociodemographic variables between the intervention and control groups, while the chi-square test was used for categorical variables. For the outcome measures (CCWS, CCAS, and CCHS), independent samples t-tests were used to compare the intervention and control groups at baseline and post-intervention, whereas paired samples t-tests were applied to assess within-group changes between pre-test and post-test measurements. In addition, repeated measures ANOVA was conducted to examine group × time interaction effects for all outcome variables. Effect sizes were calculated using Cohen’s d.

## Results

When pregnant women in the intervention and control groups were compared in terms of individual characteristics such as age, education, length of marriage, spouse’s age, family type, employment status and economic situation, no significant differences were found between the groups (*p* > 0.05). Similarly, when the women in both groups were compared in terms of obstetric characteristics such as gestational age, number of pregnancies, history of miscarriage, history of abortion, and whether the pregnancy was planned, no statistically significant differences were found between the groups (*p* > 0.05) (Table 1).

**Table 1:** Individual and obstetric characteristics of the pregnant women (N=154)

Characteristics	Intervention Group (n = 77)	Control Group (n=77)	Test/p Value
	Mean±SD	Mean±SD	
Age (years)	28.49±6.55	27.99±6.85	t = 0.469p = 0.640
Duration of Education (years)	11.61±2.98	11,52±2,64	t = 0.200p = 0.842
Duration of Marriage (years)	5.04±1.64	5.20±1.91	t = -0.320p = 0.749
Spouse’s Age (years)	30.77±6.88	28.97±1.57	t =-1.652p = 0.101
Gestational Age (weeks)	20.40±7.11	21.77±6.17	t =-1.269p = 0.206
Number of Pregnancies	1.79±0.76	1.83±0.83	t =-0.302p = 0.763
Employment Status	n (%)	n (%)	
Employed	19 (24.7)	18 (23.4)	χ² = 0.000^a^p = 1.000
Unemployed	58 (75.3)	59 (76.6)	
Family type			χ² = 0.027^a^p = 0.869
Nuclear Family	32 (41.6)	30 (39.0)	
Extended Family	45 (58.4)	47 (61.0)	
Economic Status			χ² = 0.041^a^p = 0.899
Income Less Than Expenses	14 (20,8)	14 (18.2)	
Income Equal/ Greater Than Expenses	61 (79.2)	63 (81.8)	
History of Miscarriage			
Yes	15 (19.5)	14 (18.2)	χ² = 0.000^a^p = 1.000
No	62 (80.5)	63 (81.8)	
History of Curettage			χ² = 0.264^a^p = 0.607
Yes	7 (9.1)	10 (13.0)	
No	70 (90.9)	67 (87.0)	
Planned Pregnancy			χ² = 0.137^a^p = 0.711
Yes	56 (72.7)	59 (76.6)	
No	21 (27.3)	18 (23.4)	

^a^Chi-square (Yates corrected), t: Independent samples t-test

When the total mean scores on the Climate Change Worry Scale, the Climate Change Hope Scale and the Climate Change Awareness Scale were compared between the intervention and control groups, a significant difference was found between the groups in the pre-test assessment (*p* < 0.05). Pregnant women in the intervention group scored significantly lower on the Climate Change Worry Scale and the Climate Change Awareness Scale compared to the control group; however, in the post-test assessments, the intervention group’s scores on the Climate Change Worry Scale, the Climate Change Hope Scale and the Climate Change Awareness Scale were found to have significantly higher mean scores compared to the control group (*p* < 0.05) (Table 2).

**Table 2:** Comparison of the mean scores of the individuals on the climate change worry scale (CCWS), climate change hope scale (CCHS) and climate change awareness scale (CCAS) (*N* = 154)

Climate Change Worry Scale (CCWS)	Intervention Group (*n* = 77)	Control Group (*n* = 77)	Test/*p* Value
	Mean ± SD (Min.- Max.)	Mean ± SD (Min.- Max.)	
Pre-test	21.10 ± 3.17 (10–27)	25.19 ± 2.87 (20–38)	t = -8.384 *p* = 0.000
Post-test	26.86 ± 3.64 (20–33)	21.69 ± 2.02 (17–28)	t = 10.867 *p* = 0.000
Test/p Value	t^1^ = -10.873 *p* = 0.000	t^1^= 4.340 *p* = 0.000	
Group × Time	-	-	F = 185.09 *p* < 0.001
Effect Size	-	-	d = 2.20
Climate Change Hope Scale (CCHS)			
Pre-test	35.07 ± 1.76 (31–40)	32.83 ± 2.81 (26–39)	t = 5.943 *p* = 0.000
Post-test	35.79 ± 2.70 (31–47)	29.41 ± 2.89 (23–38)	t = 14.132 *p* = 0.000
Test/p Value	t^1^ = -2.220 *p* = 0.029	t^1^= 8.439 *p* = 0.000	
Group × Time	-	-	F = 169.34 *p* < 0.001
Effect Size	-	-	d = 1.48
Climate Change Awareness Scale (CCAS)			
Pre-test	112.18 ± 15.18 (98–176)	128.94 ± 10.50 (103–148)	t = -7.960 *p* = 0.000
Post-test	137.84 ± 24.37 (11–234)	132.64 ± 10.56 (105–156)	t = 1.996 *p* = 0.049
Test/p Value	t^1^ = -18.068 *p* = 0.000	t^1^= -2.301 *p* = 0.024	
Group × Time	-	-	F = 64.300 *p* < 0.001
Effect Size	-	-	d = 1.30

^t^Independent samples t-test;

t^1^: Paired samples t-test

F: Repeated measures ANOVA; d: Cohen’s d (effect size)

Furthermore, in the post-test evaluations, it was found that the intervention group’s mean total scores on the Climate Change Worry Scale, the Climate Change Hope Scale and the Climate Change Awareness Scale were significantly higher than their pre-test means (*p* < 0.05). Furthermore, in the post-test measurements, it was found that the mean total scores of the control group on the Climate Change Worry Scale and the Climate Change Hope Scale were significantly lower than the pre-test means, whilst the mean total scores on the Climate Change Awareness Scale were significantly higher (*p* < 0.05). Furthermore, the repeated measures ANOVA conducted on Climate Change Worry Scale scores between the intervention and control groups revealed that the interaction between the group and time factors was statistically significant (F = 185.09, *p* < 0.001). This finding indicates that the change over time differed between the groups. The effect size calculated based on within-group change (Cohen’s d = 2.20) indicates that the change observed in the intervention group was of a very large magnitude (Table 2).

Similarly, the results of the repeated measures ANOVA conducted between the intervention and control groups for the Climate Change Hope Scale (F = 169.34, *p* < 0.001) and the Climate Change Awareness Scale (F = 64.300, *p* < 0.001), the results of the repeated measures ANOVA revealed that the interaction between the group and time factors was statistically significant. It was determined that the change over time in the intervention group was significantly higher than that in the control group. When considering within-group changes, the effect size for the Climate Change Hope Scale (Cohen’s d = 1.48) and the Climate Change Awareness Scale (Cohen’s d = 1.30) were found to be very large, indicating that the intervention had a very large effect (Table 2).

## Discussion

Climate change is recognized as a global issue that poses significant health risks for pregnant women, fetuses, and newborns, placing pregnant women among the most vulnerable groups. In this context, enhancing pregnant women’s awareness of climate change is of critical importance for protecting maternal and fetal health (19). However, previous studies indicate that pregnant women generally have limited and insufficient knowledge and awareness regarding climate change, highlighting a clear need for educational interventions (3, 20). A qualitative study conducted with pregnant women also emphasized that participants lacked adequate knowledge about the health impacts of climate change and that education is essential to improve awareness (20).

An experimental study examining the impact of climate change education on pregnant women demonstrated significant improvements in awareness, knowledge, and perception levels following the intervention. Moreover, the study reported that the educational intervention contributed not only to cognitive outcomes but also to individuals’ ability to evaluate environmental risks (3). Similarly, in the present study, a significant increase in climate change awareness was observed among pregnant women in the intervention group after the educational program. These findings are consistent with previous research indicating that educational interventions are effective in enhancing awareness among pregnant women (3, 21).

In parallel with the increase in awareness, a noticeable rise in anxiety levels was also observed among pregnant women. Studies conducted in the general population suggest that increased knowledge and awareness of climate change can lead individuals to perceive environmental threats more realistically, which may, in turn, elevate anxiety levels (22). This phenomenon is often explained by the concept of “eco-anxiety,” where heightened cognitive awareness of environmental issues is accompanied by emotional responses (22, 23). However, findings regarding the effects of information exposure are not entirely consistent. For instance, a study conducted in Bolivia reported no significant relationship between information exposure and climate change perceptions (24), whereas studies from India and Japan found that exposure to climate-related media content increased levels of concern (25, 26). A review by Lawrance et al. (2022) suggests that while increased awareness may elevate anxiety, it can also strengthen motivation for environmental action (7). Therefore, the increase in anxiety observed in this study may not reflect a negative outcome of the education itself, but rather a deeper understanding of risks and a more realistic evaluation of the issue.

Importantly, increased awareness appears not only to elevate anxiety levels but also to activate coping mechanisms related to environmental challenges. Li and Monroe (2019) reported that higher levels of hope enhance individuals’ motivation to generate solutions and encourage active engagement with environmental issues (27). Similarly, Ojala (2023) emphasized that educational approaches can strengthen individuals’ sense of control and foster “constructive hope” (8). In this study, the significant increase in hope levels observed in the intervention group represents a key finding. This suggests that education not only raises awareness but also supports solution-oriented thinking. Conversely, the decrease in anxiety and hope levels observed over time in the control group is another noteworthy result. Studies by Işık et al. (2025) and Bezgin and Akgül Kartal (2026) indicate that pregnant women are psychologically vulnerable to climate change and may experience anxiety, helplessness, and hopelessness in the absence of supportive interventions (1, 4). This finding suggests that without any intervention, pregnant women’s psychological well-being may decline over time in the face of climate change.

Furthermore, the large effect sizes observed in this study (d > 1) demonstrate that the educational intervention had a strong impact on both awareness and psychological outcomes among pregnant women. This finding supports the view highlighted by the Intergovernmental Panel on Climate Change (2023), which identifies educational interventions as a critical tool in addressing climate change (9). Education not only facilitates knowledge acquisition but also enhances psychological resilience, enabling individuals to develop more conscious and sustainable behavioral responses to environmental risks. For pregnant women, who are considered a vulnerable group, climate change education programs may play a dual role by increasing awareness and promoting active, solution-oriented engagement with environmental challenges.

### Strengths and limitations of the study

This study was designed as a randomized controlled trial, conducted among a vulnerable and important population group such as pregnant women, and provided a comprehensive perspective on the mental and psychological outcomes of climate change—such as awareness, anxiety, and hope— however, it has some limitations despite the use of a literature-based, structured educational intervention. First, although the study was designed as a randomized controlled trial to minimize bias, the homogeneity of the sample may limit the generalizability of the findings to a broader population of pregnant women. Additionally, since the study was conducted in a single public hospital in Istanbul, the findings may have limited generalizability to pregnant women in different regions, healthcare systems, and cultural contexts. Second, outcome assessments were conducted immediately following the intervention; this does not allow for an evaluation of the long-term effects of the education program. Third, unmeasured contextual or psychological factors in the controlled research setting where the study was conducted—as opposed to routine clinical practice—may have influenced participants’ responses and contributed to stronger observed outcomes or higher effect sizes. Additionally, although allocation to intervention and control groups was performed using a computer-based randomization system, ensuring that the researchers were not involved in group assignment, the data collection instruments relied on participants’ self-reports. Therefore, responses may have been influenced by participants’ perceptions and subjective evaluations. Despite these limitations, the study provides significant evidence regarding the effectiveness of climate change education in increasing levels of awareness, anxiety, and hope among pregnant women.

## Conclusion

This study demonstrates that climate change education programs for pregnant women have significant and strong effects on awareness, knowledge, and psychological outcomes. The educational intervention increased awareness levels, supported solution-oriented thinking, and enabled participants to perceive environmental risks more realistically. The large effect sizes observed further highlight the strength of the intervention. In this context, climate change education for pregnant women can be considered an important strategy for protecting maternal and fetal health, as it both enhances awareness and promotes solution-oriented behaviors. These findings may also provide valuable guidance for the design of future intervention programs.

Importantly, midwives and nurses play a critical role in implementing such educational interventions. As frontline healthcare professionals, they can enhance maternal awareness of climate change, provide counseling on environmental health risks, and support psychological well-being during pregnancy. Therefore, integrating climate change education into routine midwifery and nursing practice, including antenatal care services such as pregnancy classes and hospital-based education programs, may strengthen both maternal and fetal health outcomes.

## Data Availability

The datasets used in the current study are available from the correspondingauthor on reasonable request.
